# Negative Correlation between Circulating CD4^+^FOXP3^+^CD127^−^ Regulatory T Cells and Subsequent Antibody Responses to Infant Measles Vaccine but Not Diphtheria–Tetanus–Pertussis Vaccine Implies a Regulatory Role

**DOI:** 10.3389/fimmu.2017.00921

**Published:** 2017-08-14

**Authors:** Jorjoh Ndure, Fatou Noho-Konteh, Jane U. Adetifa, Momodou Cox, Francis Barker, My Thanh Le, Lady C. Sanyang, Adboulie Drammeh, Hilton C. Whittle, Ed Clarke, Magdalena Plebanski, Sarah L. Rowland-Jones, Katie L. Flanagan

**Affiliations:** ^1^Infant Immunology Group, Vaccines and Immunity Theme, MRC Unit, Fajara, Gambia; ^2^Department of Clinical Research, London School of Hygiene and Tropical Medicine, London, United Kingdom; ^3^Department of Immunology and Pathology, Monash University, Prahran, VIC, Australia; ^4^Nuffield Department of Medicine, University of Oxford, Oxford, United Kingdom

**Keywords:** vaccines, regulatory T cells, sex, beta-2 microglobulin, cytokines, immune activation, antibodies

## Abstract

Regulatory T cells (Tregs) play a key homeostatic role by suppressing immune responses. They have been targeted in mouse and human cancer studies to improve vaccine immunogenicity and tumor clearance. A number of commercially available drugs and experimental vaccine adjuvants have been shown to target Tregs. Infants have high numbers of Tregs and often have poor responses to vaccination, yet the role Tregs play in controlling vaccine immunogenicity has not been explored in this age group. Herein, we explore the role of CD4^+^FOXP3^+^CD127^−^ Tregs in controlling immunity in infant males and females to vaccination with diphtheria–tetanus–whole cell pertussis (DTP) and/or measles vaccine (MV). We find correlative evidence that circulating Tregs at the time of vaccination suppress antibody responses to MV but not DTP; and Tregs 4 weeks after DTP vaccination may suppress vaccine-specific cellular immunity. This opens the exciting possibility that Tregs may provide a future target for improved vaccine responses in early life, including reducing the number of doses of vaccine required. Such an approach would need to be safe and the benefits outweigh the risks, thus further research in this area is required.

## Introduction

The human immune system has evolved multiple mechanisms to ensure the induction of protective immunity, whilst regulating damage from over exuberant or uncontrolled immune responses. Specialized T cell subsets known as regulatory T cells (Tregs) play a vital role in this process by restoring and maintaining a homeostatic environment following an inflammatory response to an immunogen (1).

Regulatory T cells were initially identified in mice as CD4^+^ T cells highly expressing CD25 (the alpha chain of the IL-2 receptor) (2); but the later discovery of the forkhead family transcription factor FOXP3 further defined Tregs, being central to their development and function (3, 4). Its importance is highlighted in Scurfy mice and humans with IPEX syndrome where FOXP3 mutation results in uncontrolled inflammation and death (5). In humans, CD25 is also expressed by activated T cells (3), and it is the CD25^hi^ subset that is suppressive (6). Low-level expression of CD127 (the IL-7 receptor) further defines functional Tregs in humans (7). Approximately 1–5% of human CD4^+^ T cells are CD4^+^FOXP3^+^ Tregs (4) and are either thymus derived natural Tregs or peripherally derived pTregs, which are indistinguishable. Tregs act by several modes of action including the induction of inhibitory cytokines, modulation of dendritic cell function, cytolysis, and metabolic disruption (8, 9).

Neonates and young children have highly adapted immune systems as compared to adults (10). Tregs in early life are highly suppressive, are present in higher frequencies than in adults, and are more naïve and less differentiated (11–14). The role of Tregs in controlling inflammation in infectious diseases remains controversial, with studies reporting both beneficial effects of limiting immunopathology and detrimental effects with suppression of protective immunity allowing pathogen survival and persistence (15). Little is known about the role Tregs play in controlling vaccine immunogenicity in animals or humans (16). There is a need to determine whether circulating Tregs at the time of vaccination interfere with vaccine-induced responses, particularly in infants who suffer the greatest burden of infectious diseases, and where vaccine immunogenicity is often poor and Treg levels high. Understanding the homeostatic role of Tregs in infant vaccination may provide strategies to improve vaccine immunogenicity in this vulnerable age group.

We investigated for the first time whether preexisting circulating CD4^+^FOXP3^+^CD127^−^ Tregs influence antibody and cellular responses following vaccination with the live measles vaccine (MV) and killed diphtheria–tetanus–whole cell pertussis (DTP) vaccine in 9-month-old Gambian infants, and further to determine whether Treg functional capacity is altered by vaccination.

## Materials and Methods

### Study Design

**Figure 1:**
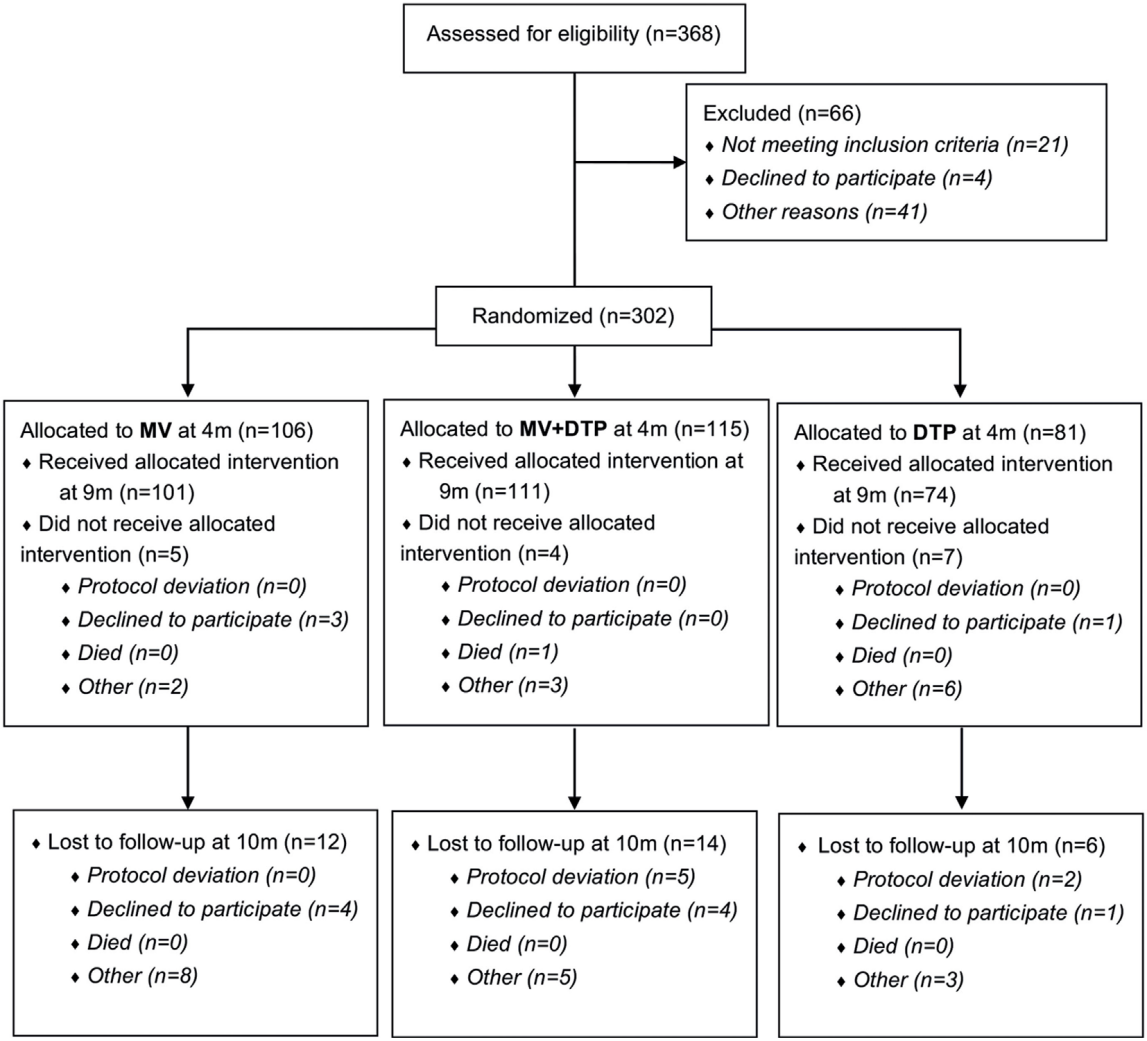
Study flowchart. 368 infants were assessed for eligibility and 302 (141 females and 161 males) randomized to one of the three vaccine groups; of whom 286 received the vaccine intervention at 9 months of age; and 32 were lost to follow-up at 10 months of age. Blood samples were collected prior to vaccination at 9 months and 4 weeks postvaccination at 10 months of age.

This study was nested into a larger randomized study investigating the immunological effects of measles vaccination, DTP vaccination, or giving both vaccines together to 9-month-old Gambian infants, some of the results of which have been published previously (17). In this study, we focused on the role that Tregs play in regulating immune responses to these vaccines. 302 infants were recruited at 4 months of age at Sukuta Health Centre, a peri-urban area 20 km from the coast of The Gambia. Eligibility criteria included being well with no history of chronic illness, apyrexial (<37.5°C), normal weight-for-age, and all recommended vaccines received to date. Infants were randomized into one of three vaccine groups (Table 1). At 4 months of age, Group 1 received DTP3 as normal while Groups 2 and 3 had their third dose of DTP withheld. All three groups received oral polio vaccine and *Haemophilus influenzae b* vaccine at 4 months of age. At 9 months of age, Group 1 were given MV alone; Group 2 received DTP3 with MV; and Group 3 received DTP3 alone (Figure 1). Males and females were randomized separately.

**Table 1:** Vaccines given from birth until 9 months of age in the three vaccine groups.

	Birth	8 weeks	12 weeks	16 weeks	9 months
MV group	BCG, OPV, HepB	DTP1, Hib, OPV, HepB	DTP2, Hib, OPV	DTP3, Hib, OPV, HepB	MV
MV + DTP group	BCG, OPV, HepB	DTP1, Hib, OPV, HepB	DTP2, Hib, OPV	Hib, OPV, HepB	MV + DTP3
DTP group	BCG, OPV, HepB	DTP1, Hib, OPV, HepB	DTP2, Hib, OPV	Hib, OPV, HepB	DTP3

*BCG, bacillus Calmette–Guérin vaccine; DTP1/2/3, diphtheria–tetanus–whole cell pertussis vaccine dose 1/2/3; HepB, hepatitis B vaccine; Hib, Haemophilus influenzae group b vaccine; MV, measles vaccine; OPV, oral polio vaccine*.

### Ethics Statement

The study protocol was approved by the Joint Gambia Government/MRC Ethics Committee (project number SCC1085) and the London School of Hygiene and Tropical Medicine Ethics Committee. Written informed consent was provided by a parent/guardian of all participating infants.

### Blood Sampling

Blood samples were taken at 9 months of age prior to vaccine administration, and 4 weeks later (10 months). Four and a half milliliters of venous blood was collected into heparin tubes and transported to the laboratory within 4 h of collection.

### *Ex Vivo* Flow Cytometric Analysis for Tregs, T Cell Memory, and Function

**Figure 2:**
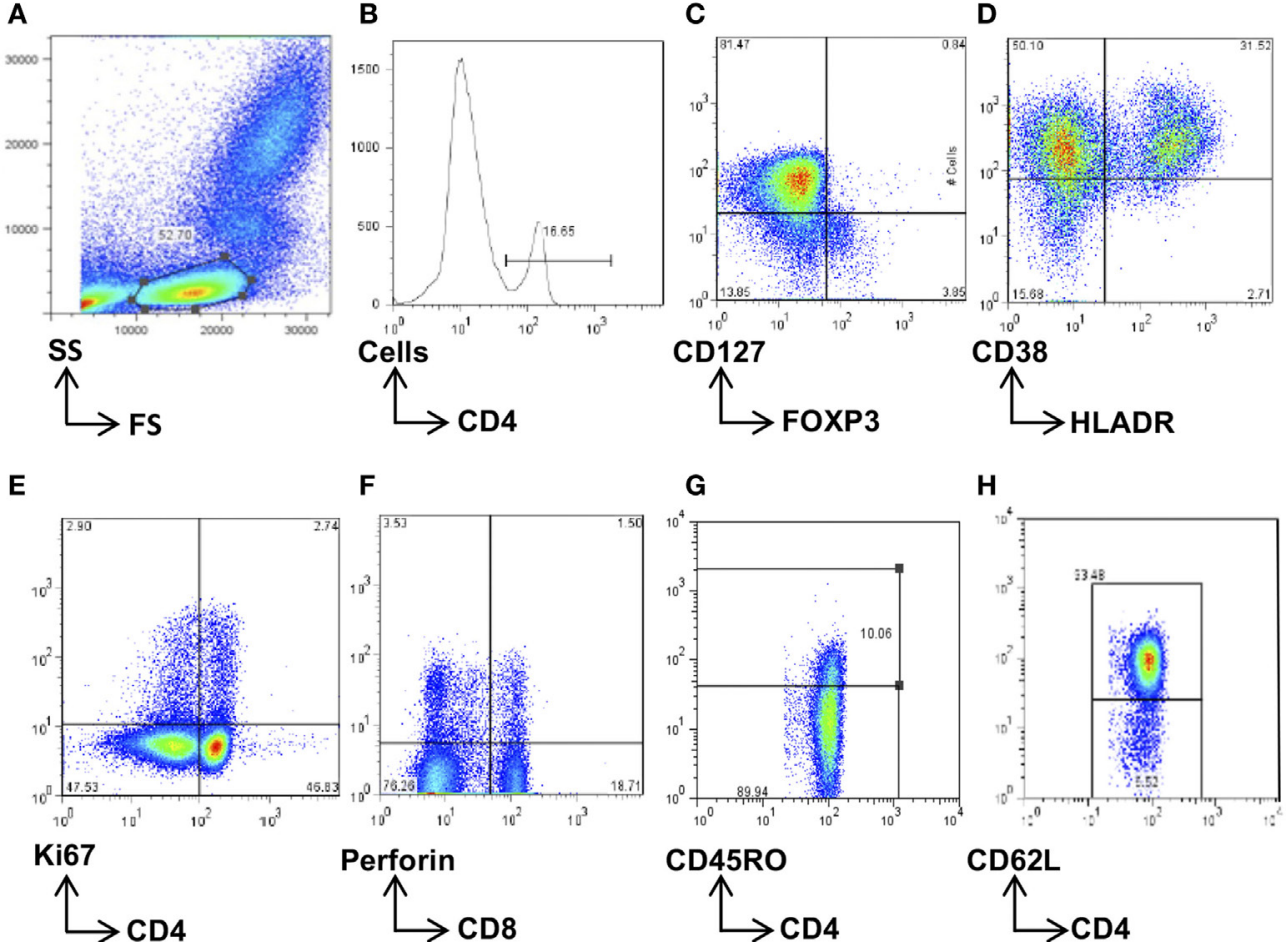
Flow cytometry gating strategy. Total lymphocytes were first gated on a forward scatter (FS)/side scatter (SS) plot **(A)** and then gated on the CD4^+^ **(B)** or CD8^+^ population. These were then further gated for the subsets of interest, namely, CD4^+^FOXP3^+^CD127^−^ regulatory T cells **(C)**, HLADR^+^CD38^+^-activated CD4^+^ or CD8^+^ cells **(D)**, Ki67^+^-proliferating CD4^+^ and CD8^+^ cells **(E)**, and perforin^+^ CD8 T cells **(F)**. The memory cells were first separated according to CD45RO expression **(G)** and then further phenotyped according to CD62L expression **(H)**. Data were analyzed using FlowJo software, and population frequencies expressed as percent of the CD4 or CD8 parent population.

50 µL whole blood was stained with cocktails of fluorochrome-conjugated surface antibodies to analyze for CD4 (CD4 APC-Cy7 or PerCP) and CD8 (CD8 Pacific blue) T cells expressing markers of Tregs (CD127 PE, FOXP3 APC); memory (CD45RO APC, CD62L PE-Cy7); terminal differentiation (CD57 FITC); activation (HLADR PerCP and CD38 PE-Cy7), proliferation (Ki67 FITC), and perforin production (Perforin PE) [Becton-Dickinson (BD) for all fluorochromes except for CD8 PB, FOXP3 APC, and CD62L PE-Cy7, which were from E-biosciences]. Red blood cells were lysed, and cells washed and incubated with surface antibodies for 30 min at 4°C. Cells for Treg analysis were then washed in 200 µL permeabilization buffer (E-biosciences); incubated with normal rat serum (1:50 dilution) for 15 min, 4°C in the dark, then FOXP3 added and incubated for 30 min at 4°C. Cells for Ki67 and perforin intracellular staining were washed with BD permeabilization buffer, Ki67 and perforin fluorochromes were added, and cells incubated for 30 min at 4°C in the dark. Stained cells were resuspended in 150 µL fix buffer (1% formalin in PBS), then acquired on the Cyan Flow cytometer (CyanADP). Flow cytometry data were analyzed using FlowJo software (Treestar, CA, USA) according to the gating strategies described (Figure 2).

### Vaccine Antibody Titers

The measles IgG hemagglutination inhibition assay (HAI) was performed using monkey red blood cells as described previously (18). Results are expressed as log_2_ units, the minimum detection level being 31.2 mIU, and a protective level defined as ≥125 mIU (log_2_ titer ≥3). A multiplex microsphere-based fluorescent immunoassay for IgG antibodies to diphtheria toxoid (Dtx), tetanus toxoid (Ttx), and four pertussis antigens [pertussis toxoid (Ptx), fimbriae, pertaxin, and filamentous hemagglutinin] was performed at the National Institute of Public Health and the Environment, Netherlands using published protocols (19). Protective levels for Dtx and Ttx are ≥0.1 IU/mL, but there is no established protective level for the pertussis antibodies.

### Beta-2 Microglobulin Assay

The plasma beta-2 microglobulin (β2m) levels in milligrams per milliliter were measured by an automated microparticle enzyme immunoassay using an AxSYM automated machine (Abbott Laboratories, Wiesbaden, Germany) according to the manufacturer’s instructions.

### Whole Blood Cultures

Heparinized whole blood was cultured in 100 µL aliquots in 96 well U-bottom plates with tetanus toxoid (TT) (10 µg/mL, Sanofi Pasteur, France); a measles peptide pool of 122 15mer peptides overlapping by 10 amino acids spanning the measles protein hemagglutinin (all 1 µg/mL final concentration, Sigma-Genosys, UK); anti-CD3 (αCD3) (5 µg/mL, BD) plus anti-CD28 (αCD28) (5 µg/mL, E-biosciences) as a positive control T cell stimulus; and medium alone as a background negative control. Antigen pulsed plates were incubated for 16 h at 37°C, 5% CO_2_, centrifuged and 50 µL of supernatant collected and stored at −20°C for cytokine analysis.

### Multiplex Cytokine Analysis

The Bio-Plex 200 Suspension Array system was used to analyze cytokines in plasma and culture supernatants (Bio-Rad, Belgium). The cytokines analyzed were interferon-gamma (IFN-γ), tumor necrosis factor (TNF), interleukin-1 beta (IL-1β), IL-4, and IL-10. Out of range values were assigned twice the upper limit of detection or half the lower limit of detection for those above and below range, respectively, as in previous studies (17). Medium background was subtracted from the antigen-stimulated value to establish antigen-specific cytokine production.

### Statistical Analysis

Differences in Treg frequencies in vaccine groups at different time points were analyzed by two-sided Mann–Whitney *U* tests. Correlations were analyzed using Spearman’s rank correlation coefficient. *p* ≤ 0.05 was considered significant, and Bonferroni corrections for multiple testing were performed. Data were analyzed using GraphPad prism version 6.0 (GraphPad software, CA, USA).

## Results

### No Convincing Effect of Vaccination on Circulating CD4^+^FOXP3^+^CD127^−^ Treg Frequencies

**Figure 3:**
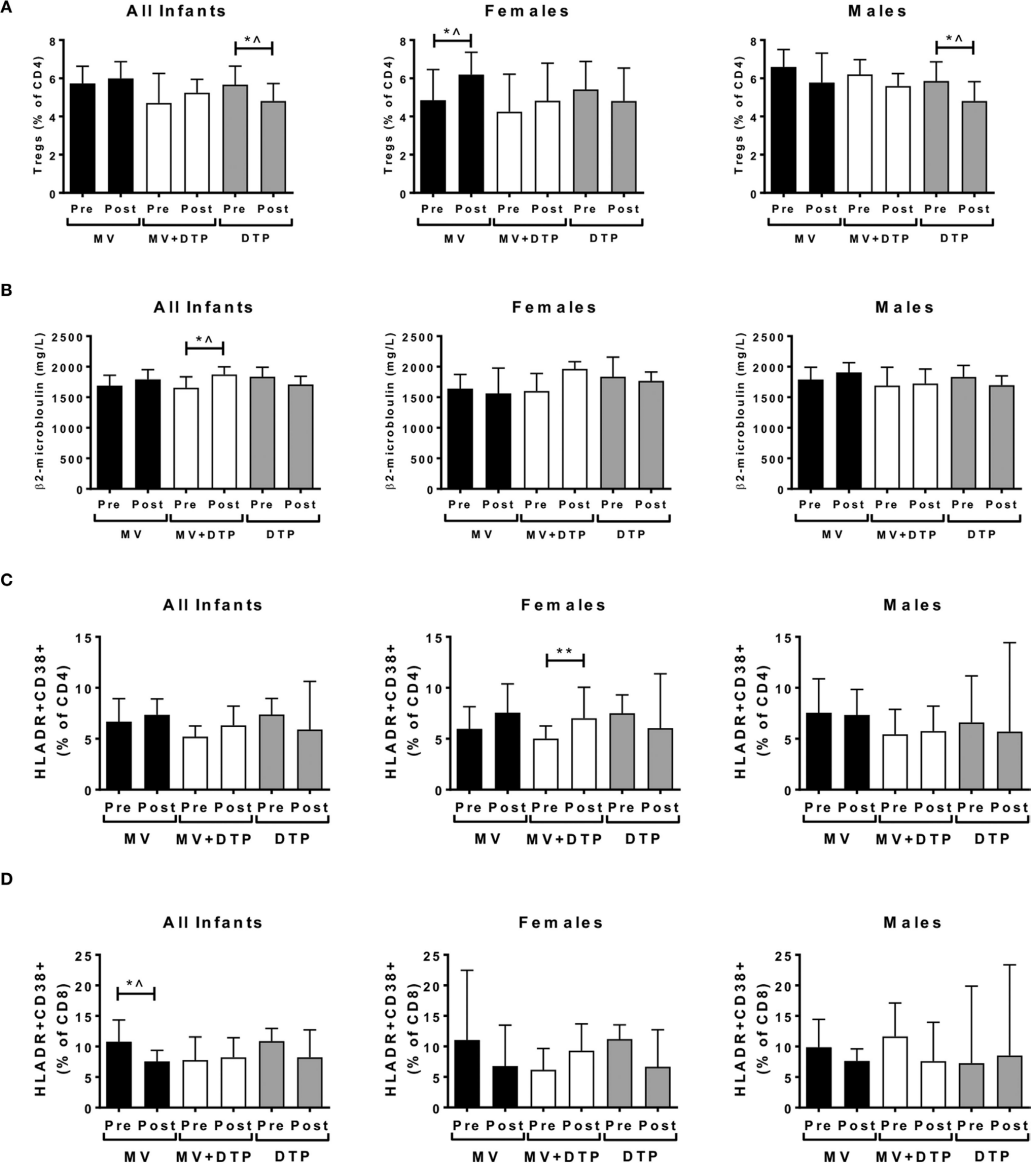
Regulatory T cells (Tregs), plasma β2m, and activated CD4 and CD8 T cell levels before and after vaccination. Results for CD4^+^FOXP3^+^CD127^−^ Treg frequencies **(A)**, plasma β2m levels **(B)**, activated CD4 T cells **(C)**, and activated CD8 T cells **(D)** in the three vaccines groups at baseline (Pre) and 4 weeks after vaccination (post) for all infants combined, females and males separately. The bars show the median value in milligrams per liter; the error bars indicate the 95% confidence interval. Data were analyzed by Mann–Whitney *U* test, *p ≤ 0.05, **p ≤ 0.01, ^, no longer significant after correcting for multiple testing. Data are shown for Tregs: *n* = 194 infants, measles vaccine (MV) group *n* = 68 (31F, 37M), MV + DTP group *n* = 77 (43F, 34M), DTP *n* = 49 (26F, 23M); β2m: *n* = 259 infants, MV group *n* = 91 (39F, 52M), MV + DTP group *n* = 99 (47F, 52M), DTP *n* = 69 (33F, 36M); activated T cells: *n* = 158 infants, MV group *n* = 61 (28F, 33M), MV + DTP group *n* = 63 (32F, 31M), DTP *n* = 34 (19F, 15M).

We first analyzed whether circulating Treg frequencies were altered following vaccination with MV, DTP, or both vaccines combined. When male and female donors were analyzed together, there was a significant decline in Tregs in the DTP-vaccinated group (*p* = 0.039), but no change in the other two groups (Figure 3A). When males and females were analyzed separately, there was an increase in circulating Tregs in measles-vaccinated females (*p* = 0.040) and a decrease in DTP-vaccinated males (*p* = 0.029) (Figure 3A). However, none of these changes remained significant after Bonferroni correcting for multiple testing and were therefore not considered convincing evidence of changes in Tregs postvaccination.

### Vaccination Affects Immune Activation and T Cell Function

**Figure 4:**
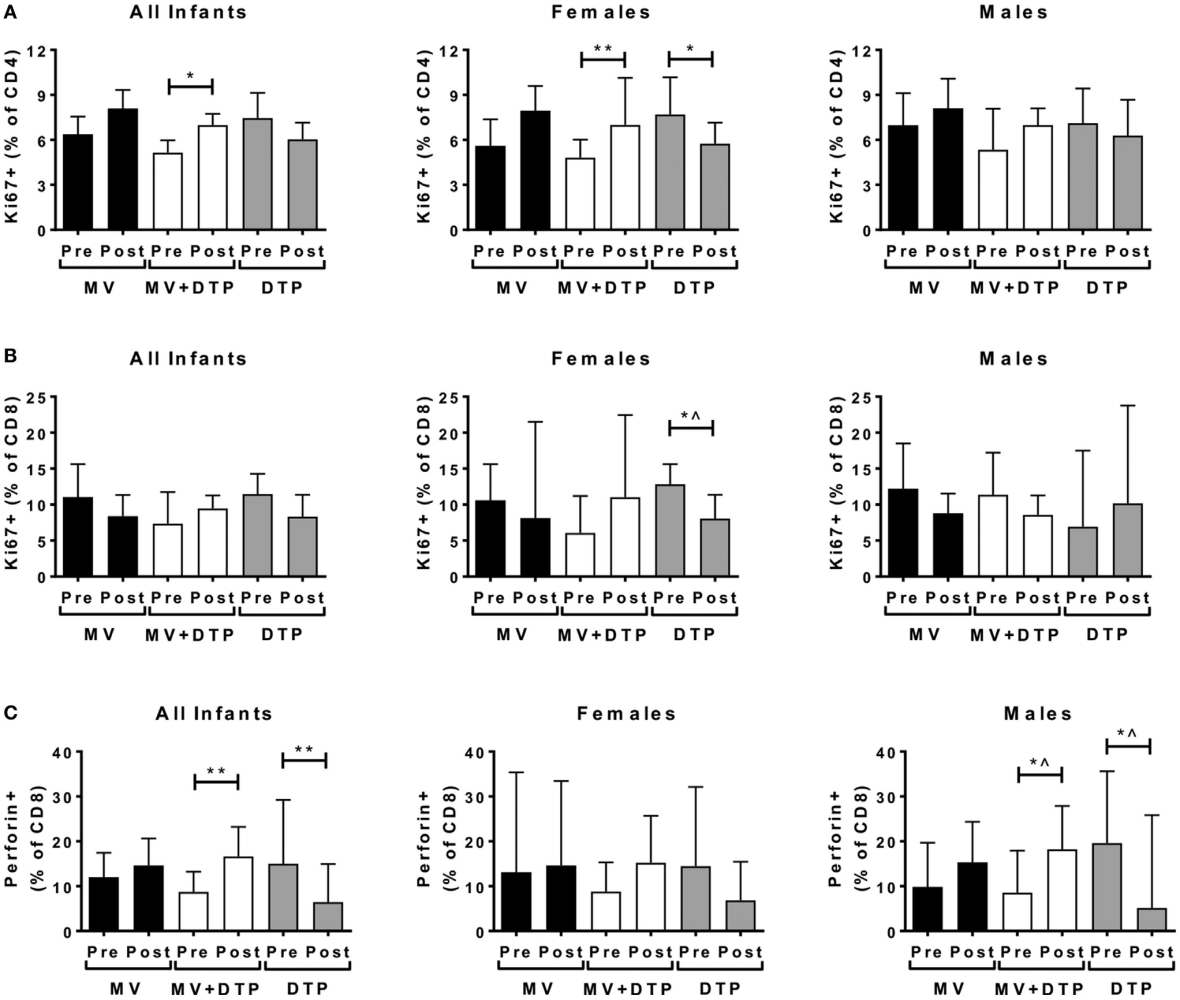
Proliferating and perforin producing T cell frequencies before and after vaccination. Results for CD4 T cell proliferation **(A)**, CD8 T cell proliferation **(B)**, and intracellular perforin levels **(C)** in the three vaccines groups at baseline (pre) and 4 weeks after vaccination (post) for all infants combined, females and males separately. The bars show the median value; the error bars indicate the 95% confidence interval. Data were analyzed by Mann–Whitney *U* test, *p ≤ 0.05, **p ≤ 0.01, ^, no longer significant after correcting for multiple testing. Data are shown for *n* = 158 infants, measles vaccine (MV) group *n* = 61 (28F, 33M), MV + DTP group *n* = 63 (32F, 31M), and DTP *n* = 34 (19F, 15M).

Beta-2 microglobulin levels increased after MV + DTP vaccination when all infants were analyzed together (*p* = 0.035), but not when analyzed by sex (Figure 3B). The frequency of CD38^+^HLADR^+^-activated CD4 T cells increased in the females who received MV + DTP simultaneously (*p* = 0.0073), but no other group (Figure 3C). By contrast CD38^+^HLADR^+^ CD8 T cells declined in infants following MV (*p* = 0.0403), but not the other vaccine groups (Figure 3D). CD4^+^ T cell proliferation determined by Ki7 expression also increased in the MV + DTP group (all infants *p* = 0.0038; females *p* = 0.0017) (Figure 4A), while levels of proliferating CD4 and CD8 T cells declined in the DTP-vaccinated females (*p* = 0.0137 and 0.0353, respectively), but not males (Figures 4A,B). After Bonferroni correction for multiple testing only the above changes in CD4 T cells proved to be statistically significant, namely, the altered activated and proliferating CD4 T cells (Figures 3C and 4A).

CD8^+^ T cell perforin production increased in the MV + DTP group (all infants *p* = 0.008; males *p* = 0.0376) but declined in the DTP-vaccinated groups (all infants *p* = 0.0077; males *p* = 0.0224) (Figure 4C). After correcting for multiple testing, males and females combined had an increase in perforin^+^ CD8 T cells after MV + DTP and a decline after DTP alone.

None of the above effector readouts of proliferation, perforin production, or immune activation correlated negatively with circulating Tregs either at baseline or 4 weeks after vaccination.

### Altered Memory T Cell Populations in Dual Vaccinated Infants

**Figure 5:**
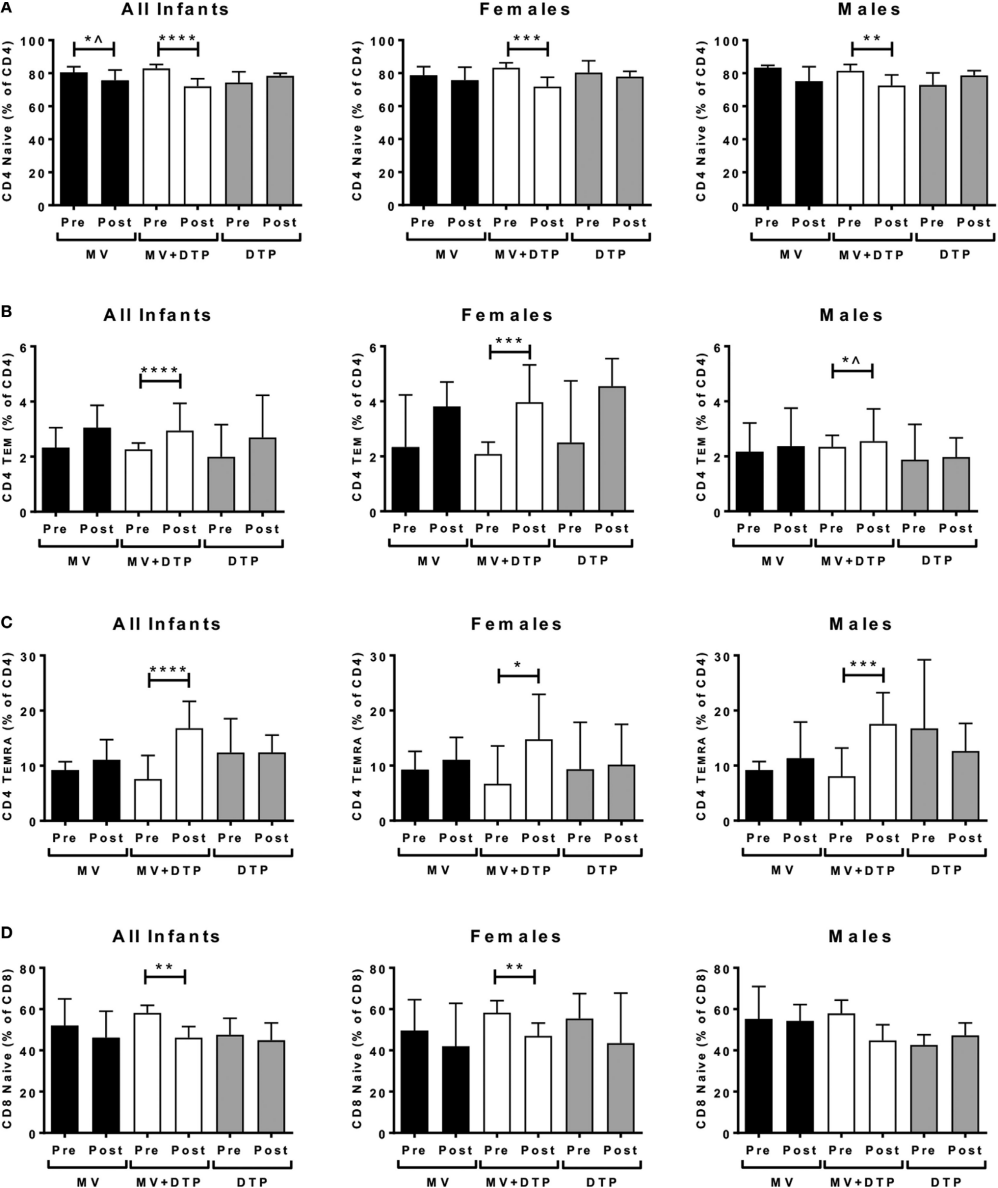
Effect of vaccination on naïve and memory T cell frequencies. Results for CD4 naïve T cell frequencies **(A)**, CD4 T_EM_ (CD4^+^CD45RO^+^CD62L^−^) cells **(B)**, CD4 T_EMRA_ (CD4^+^CD45RO^−^CD62L^−^) cells **(C)**, and CD8 naïve T cell frequencies **(D)** in the three vaccines groups at baseline (pre) and 4 weeks after vaccination (post) for all infants combined, females and males separately. The bars show the median value; the error bars indicate the 95% confidence interval. Data were analyzed by Mann–Whitney *U* test, *p ≤ 0.05, **p ≤ 0.01, ***p ≤ 0.001, ****p ≤ 0.0001, ^, no longer significant after correcting for multiple testing. Data are shown for *n* = 230 infants, measles vaccine (MV) group *n* = 83 (37F, 46M), MV + DTP group *n* = 91 (43F, 48M), and DTP *n* = 56 (27F, 29M).

We analyzed for effects on *ex vivo* circulating memory T cell populations. It was striking that the only significant changes occurred in the group that received the MV and DTP vaccines at the same time, with no changes in the single vaccine groups. The CD4 naïve (CD4^+^CD45RO^−^CD62L^+^) population declined in the MV + DTP group for all infants (*p* < 0.0001), males (*p* < 0.0024), and females (*p* < 0.0001) (Figure 5A); while the T cell effector memory (T_EM_) (CD4^+^CD45RO^+^CD62L^−^) and CD45RA^+^ effectors (T_EMRA_) (CD4^+^CD45RO^−^CD62L^−^) increased in this group (T_EM_: all infants *p* = 0.0001, females *p* = 0.0006, males *p* = 0.0337; T_EMRA_: all infants *p* < 0.0001, females *p* = 0.012, males *p* = 0.0009) (Figures 5B,C). The decreased CD4 naïve T cell frequency in MV infants (*p* = 0.0292) was not significant after correcting for multiple testing (Figure 5A), nor was the T_EM_ increase in males (Figure 5B). The CD8 population was less affected with only the naïve subset declining significantly in the MV + DTP group for all infants (*p* = 0.0038) and females (*p* = 0.0087) (Figure 5D). There was no change in central memory (T_CM_) (CD45RO^+^CD62L^+^) frequencies for CD4 or CD8 T cells for any vaccine group. The frequency of terminally differentiated T cells was examined by CD57 expression, hinting at a decline in MV + DTP-vaccinated infants for both CD4 and CD8 populations (*p* = 0.0455 and 0.0464, respectively), although neither were significant after correcting for multiple testing (not shown).

### Baseline CD4^+^FOXP3^+^CD127^−^ Tregs Negatively Correlate with Antibody Responses to MV but Not DTP

**Figure 6:**
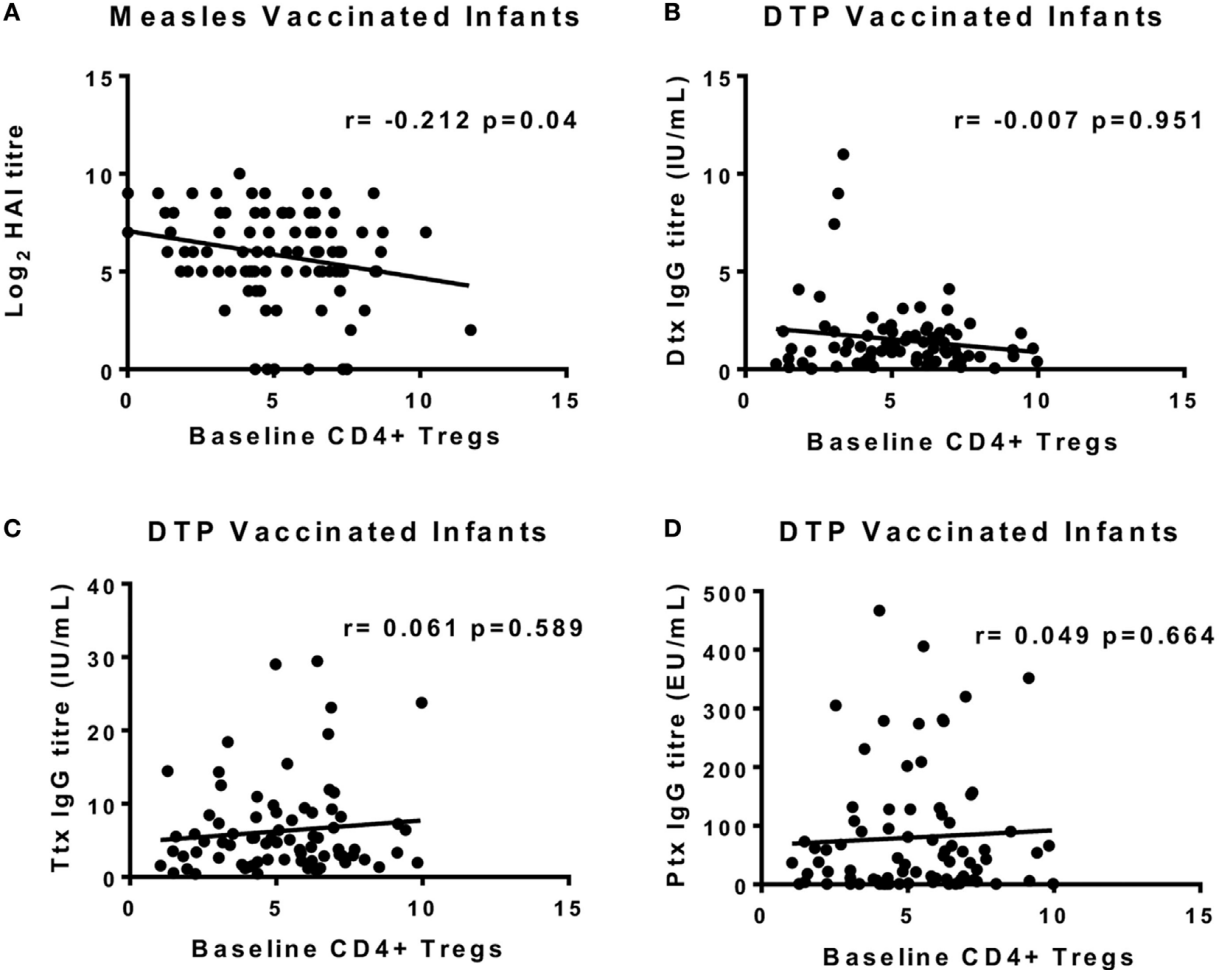
Correlations between baseline circulating regulatory T cells (Tregs) at the time of vaccination and vaccine antibodies 4 weeks later. The frequency of Tregs at 9 months of age on the day of vaccination is shown on the *x*-axis, and the IgG antibody titers at 10 months are shown on the *y*-axis. **(A)** There was a significant inverse correlation between baseline Tregs and measles HAI titers for all measles-vaccinated infants [measles vaccine (MV) and MV + DTP groups combined]. Diphtheria toxoid (Dtx) titers **(B)**, Ttx titers **(C)**, and pertussis toxoid (Ptx) titers **(D)** for DTP vaccine recipients (DTP and MV + DTP groups) failed to correlate with baseline Tregs. The line indicates the best-fit correlation using Spearman’s rank correlation coefficient, and the correlation coefficient (*r* value) and *p* value for the correlation are shown. Data for 97 measles-vaccinated (47F, 50M) and 81 DTP-vaccinated (45F, 36M) infants are shown.

We previously showed that measles antibody titers 4 weeks after vaccination were not affected by simultaneous administration of DTP; nor were tetanus toxoid (Ttx), diphtheria toxoid (Dtx), and Ptx titers post-DTP vaccination altered by giving MV at the same time (17). To increase statistical power, we combined the MV and MV + DTP group to analyze for measles Ab correlations between baseline circulating Tregs and vaccine antibody levels 4 weeks later; and the DTP and MV + DTP groups to analyze for DTP Ab correlations. This showed a significant negative correlation between baseline Tregs and measles Ab titers in infants who received MV (MV and MV + DTP groups combined) (*r* = −0.208, *p* = 0.048) (Figure 6A); but no correlation with any of the antibody readouts for DTP-vaccinated infants (Figures 6B–D). There were no significant correlations between Tregs and vaccine antibodies when males and females were analyzed separately.

### No Correlations between Baseline Tregs and Postvaccination Cellular Responses

We could not perform functional Treg assays in this study for logistic reasons including the multiple assays being conducted, small blood volumes, and lack of flow cytometry cell sorting facilities in The Gambia. We thus chose to analyze for correlations between CD4^+^FOXP3^+^CD127^−^ Tregs and readouts of vaccine-specific cellular immunity. We questioned whether baseline Tregs on the day of vaccination correlated negatively with subsequent vaccine-specific pro-inflammatory (IL-1β, TNF), Th1 (TNF, IFN-γ) or Th2 (IL-4) cellular responses 2 weeks after vaccination, or with the immunosuppressive cytokine IL-10, which can also be produced by activated Th1 and Th2 cells. There was no evidence for any significant correlation between baseline Tregs and subsequent cytokine responses to measles peptide or TT stimulation to suggest an immunoregulatory role for circulating Tregs at the time of vaccination.

### Functionally Suppressive Tregs Post-DTP Vaccination

**Figure 7:**
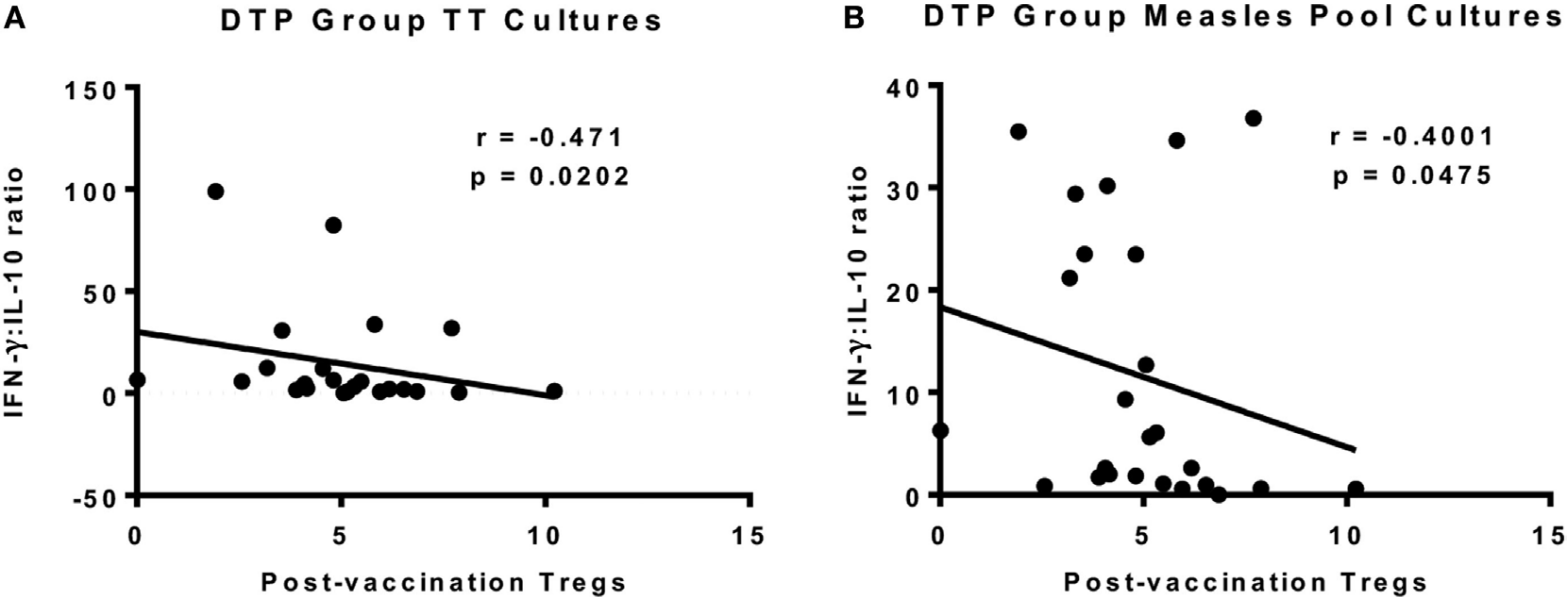
Correlation between regulatory T cells (Tregs) and vaccine-specific cytokine responses 4 weeks after vaccination. There was a significant inverse correlation between postvaccination Tregs and interferon-gamma (IFN-γ):IL-10 in TT culture supernatants at the same time point for DTP-vaccinated infants **(A)**. There was also a significant inverse correlation for IFN-γ:IL-10 in measles peptide pool cultures in the DTP group **(B)**. The line indicates the best-fit correlation using Spearman’s rank correlation coefficient, and the correlation coefficient (*r* value) and *p* value for the correlation are shown. Data for 25 DTP-vaccinated infants (18F, 7M) are shown.

We next analyzed for a correlation between the circulating Tregs 4 weeks postvaccination and *in vitro* cytokine responses to the measles peptide pool, TT, PPD, and αCD3/αCD28 at the same time point. There was a negative correlation between postvaccination Tregs and the IFN-γ:IL-10 ratio in TT cultures (*p* = 0.0202, *r* = −0.471) and measles peptide cultures (*p* = 0.0475, *r* = −0.4001) in the DTP group only (Figures 7A,B). There was no correlation between postvaccination Tregs and cytokine responses to the T cell stimulus αCD3/αCD28 or the unrelated antigen PPD for any vaccine group to support an immunoregulatory role.

## Discussion

Several studies suggest that FOXP3^+^ Tregs suppress the generation of immune responses to vaccination (16). The DEREG mouse model is a useful tool in this respect allowing Tregs depletion *in vivo* (20). Treg depletion using this model showed enhanced vaccine antitumor responses in a melanoma vaccine trial (21) and enhanced vaccine-induced solid tumor immune responses (22). In the latter study, Treg depletion was associated with enhanced CD8 activation and IFN-γ production, and increased NK cells. In mice, the administration of anti-CD25 mAbs to deplete Tregs enhanced CD4 and CD8 T cell responses to BCG and hepatitis B vaccines (23), and preexisting CD4^+^CD25^+^ Tregs suppressed BCG responses *in vitro* and *in vivo* (24).

Little is known about the role of Tregs in controlling human vaccine responses for any age group. Furthermore, translating the mouse into human studies may not be appropriate since their Tregs are phenotypically distinct. A human study showed marked increases in CD4^+^CD25^hi^FOXP3^+^ Tregs following vaccination with a DC-based HIV vaccine, and enhanced T cell immunity *in vitro* following Treg depletion (25). We have previously shown that infant BCG vaccination induces CD4^+^CD25^+^FOXP3^+^ Tregs, but they did not negatively correlate with IFN-γ reactivity to PPD to support a regulatory role (26). By contrast, the TB vaccine MVA85A was associated with a small decrease in CD4^+^CD25^hi^CD39^+^ Tregs (27) decreased TGF-β mRNA and serum levels up to 12 weeks after vaccination (28).

The changes in Treg frequencies after vaccination were not significant after correcting for multiple testing, thus the question of whether MV or DTP vaccination alters Treg frequencies remains unresolved and further studies are needed. Tregs remained stable in the group that received MV + DTP; but this group had increased CD8 T cell perforin production; and increased CD4 T cell activation and proliferation among females but not males. The MV + DTP group also experienced a decline in naïve CD4 and CD8 T cell frequencies, and increased CD4 T_EM_ and CD4 T_EMRA_ populations following vaccination, with some sex differences in this effect. These results are consistent with our previous findings that combining MV with DTP vaccination has immune suppressing effects on PPD cellular responses and RNA expression in males, but the opposite effect in females, compared to giving DTP or MV alone (17).

Sex differences have been described for antibody and cellular responses to many vaccines, with females generally mounting higher responses, and also suffering greater adverse events (29–32). Females are also generally more susceptible to the non-targeted heterologous effects of vaccines, whereby vaccination alters the host’s response to subsequent exposure to unrelated (heterologous) organisms or vaccines (33, 34). Thus the sex differences described in this study are to be expected, but are often overlooked in immunological studies because they are not specifically analyzed for. Sex differences in Treg frequencies have not be described in infants although males have higher levels later in life (32), and we found no convincing evidence of a sex difference in our study either before or after vaccination.

In a small study, baseline regulatory cytokine gene expression (TGF-β and IL-10) at the time of vaccination with the malaria vaccines RTS,S/ASO2A and MVA-CS showed an inverse correlation with subsequent antibody responses (35). Our results similarly suggest that Tregs at the time of vaccination suppress primary antibody responses to measles vaccination, but had no effect on secondary DTP vaccine antibody responses. We have since repeated this finding of a significant negative correlation between baseline Tregs and MV antibodies using a different Treg definition (CD4^+^CD25^hi^FOXP3^+^) and different time point (2 weeks post-measles vaccination) in a separate prospective cohort (in preparation). Potential mechanisms include direct suppression of B cells *via* cell-contact mechanisms involving TGF-β and CTLA-4 (36); and suppression of T cell help essential for B cell activation and expansion (37). If confirmed, Tregs could provide a functional target for enhancing vaccine antibody responses in infancy.

We analyzed for associations between Tregs and key CMI readouts as an indication of Treg function. We hypothesized that baseline Tregs at vaccination might suppress subsequent CMI to vaccination, and postvaccination Tregs might be functionally suppressive. We further speculated that the live and killed vaccines might have different effects in these respects. We found no evidence that baseline Tregs affected subsequent *ex vivo* plasma cytokines or β2m, activated or proliferating CD4 or CD8 T cells, CD8 T cell perforin production, or measles and TT-specific cytokine responses.

The inverse correlation between the circulating CD4^+^FOXP3^+^CD127^−^ Tregs post-DTP vaccination and the postvaccination IFN-γ:IL-10 ratio in TT and measles peptide cultures, but not PPD or αCD3/αCD28, may support a vaccine-specific immunoregulatory role. No such correlations were observed in the MV or MV + DTP groups. However, given the multiple factors analyzed in these correlations these findings may well be type 1 error and further studies are required. If true, it would suggest that some vaccines, but not others, may induce suppressive Tregs. Indeed, we have found that mice immunized with aluminum adjuvant preferentially expand CD4^+^CD25^+^FOXP3^+^TNFR2^+^ Tregs in draining lymph nodes, providing a potential mechanism whereby Tregs may be more functionally suppressive following vaccination with the aluminum adjuvanted DTP vaccine (unpublished). We previously showed suppressed type 1 immunity to αCD3/αCD28 stimulation in DTP-vaccinated females but not males in this cohort (17); but found no significant correlations between Tregs and αCD3/αCD28 cytokine responses to suggest Tregs were responsible for this.

There are several limitations to this study. We only had one postvaccination time point that does not reveal the dynamics of the immune factors analyzed. Power was reduced due to multiple comparisons, but corrections for multiple testing were done to allow for this. The Treg function conclusions are based on correlations rather than functional suppressive assays, although many studies use this approach. Nevertheless the results offer potential insights into the immunoregulatory role that circulating CD4^+^FOXP3^+^CD127^−^ Tregs, either at the time of vaccination or postvaccination, may play in controlling vaccine immunogenicity in infants.

Certain vaccine adjuvants preferentially expand Teff over Tregs, e.g., the TLR3 agonist Poly(I:C) and the TLR9 agonist CpG-ODN; whereas others favor Treg expansion, e.g., the TLR7 agonist imiquimod (38). Thus adjuvants might be selected for future infant vaccines that allow for optimal Teff responses. Chemokine receptor 4 (CCR4) antagonists have been used as vaccine adjuvants to target and decrease local recruitment of CCR4^+^ Tregs to amplify vaccine responses at the immunization site (39). The mAb to OX40, part of the TNFR superfamily, increases Teff function while blocking Treg function; and humanized OX40 clones have been generated which enhance the immunogenicity of vaccines against infectious diseases (40). There are also a number of therapeutic agents that can manipulate Tregs *in vivo*. For example, low dose cyclophosphamide transiently decreases Treg frequencies while preserving effector T cell (Teff) function, permitting enhanced vaccine immunogenicity in mouse and human cancer vaccine trials (41, 42). Treg depletion with anti-CD25 monoclonal antibodies enhanced vaccine efficacy in mouse melanoma (43) and pancreatic carcinoma (44). The antihuman CD25 mAbs basiliximab and daclizumab decrease Treg number and function by blocking IL-2 signaling (45, 46); and daclizumab has been used to deplete Tregs and improve effector responses in human breast cancer vaccine trials (47, 48). The human mAb, ipilimumab, inhibits Tregs by blocking CTLA-4 and is FDA approved for use in melanoma patients (49).

While the above therapeutic approaches are not currently appropriate for infant vaccination strategies where any immune benefits will likely be outweighed by adverse effects, they demonstrate the future potential for Treg manipulation to improve vaccine immunogenicity. Transient Treg depletion would be preferable, since prolonged depletion could lead to increased immune pathology and autoimmunity. By contrast, the adjuvant approaches discussed above are already available and could be used to improve vaccine immunogenicity in vulnerable populations such as neonates and infants. To do this, we need to understand the role that Tregs play in controlling responses to early life vaccines. We hope our results will galvanize further research in this dynamic and evolving field.

## Ethics Statement

The study protocol was approved by the Joint Gambia Government/MRC Ethics Committee (project number SCC1085) and the London School of Hygiene and Tropical Medicine Ethics Committee. Written informed consent was provided by a parent/guardian of all participating infants.

## Author Contributions

HW, KF, and SR-J designed the study; JA oversaw infant recruitment/vaccination/bleeding/clinical assessment; JN, FN-K, MC, FB, ML, LS, and AD did the laboratory assays; JN, KF, MP, and EC did the analysis and interpreted the data; all the authors critically revised and approved the manuscript and are accountable for the accuracy and integrity of the work.

## Conflict of Interest Statement

The authors do not have a commercial or other association that might pose a conflict of interest.

## References

[1] Belkaid Y. Regulatory T cells and infection: a dangerous necessity. Nat Rev Immunol (2007) 7(11):875–88. 10.1038/nri2189 17948021

[2] Sakaguchi S Sakaguchi N Asano M Itoh M Toda M. Immunologic self-tolerance maintained by activated T cells expressing IL-2 receptor alpha-chains (CD25). Breakdown of a single mechanism of self-tolerance causes various autoimmune diseases. J Immunol (1995) 155(3):1151–64. 7636184

[3] Fontenot JD Gavin MA Rudensky AY. Foxp3 programs the development and function of CD4+CD25+ regulatory T cells. Nat Immunol (2003) 4(4):330–6. 10.1038/ni904 12612578

[4] Hori S Nomura T Sakaguchi S. Control of regulatory T cell development by the transcription factor Foxp3. Science (2003) 299(5609):1057–61. 10.1126/science.1079490 12522256

[5] Bennett CL Christie J Ramsdell F Brunkow ME Ferguson PJ Whitesell L The immune dysregulation, polyendocrinopathy, enteropathy, X-linked syndrome (IPEX) is caused by mutations of FOXP3. Nat Genet (2000) 27(1):20–1. 10.1038/83713 11137993

[6] Vukmanovic-Stejic M Agius E Booth N Dunne PJ Lacy KE Reed JR The kinetics of CD4+Foxp3+ T cell accumulation during a human cutaneous antigen-specific memory response in vivo. J Clin Invest (2008) 118(11):3639–50. 10.1172/JCI135834 18924611 PMC2556297

[7] Liu W Putnam AL Xu-Yu Z Szot GL Lee MR Zhu S CD127 expression inversely correlates with FoxP3 and suppressive function of human CD4+ T reg cells. J Exp Med (2006) 203(7):1701–11. 10.1084/jem.20060772 16818678 PMC2118339

[8] Vignali D. How many mechanisms do regulatory T cells need? Eur J Immunol (2008) 38(4):908–11. 10.1002/eji.200738114 18395857

[9] Vignali DA Collison LW Workman CJ. How regulatory T cells work. Nat Rev Immunol (2008) 8(7):523–32. 10.1038/nri2343 18566595 PMC2665249

[10] Levy O. Innate immunity of the newborn: basic mechanisms and clinical correlates. Nat Rev Immunol (2007) 7(5):379–90. 10.1038/nri2075 17457344

[11] Flanagan KL Halliday A Burl S Landgraf K Jagne YJ Noho-Konteh F The effect of placental malaria infection on cord blood and maternal immunoregulatory responses at birth. Eur J Immunol (2010) 40(4):1062–72. 10.1002/eji.200939638 20039298

[12] Fujimaki W Takahashi N Ohnuma K Nagatsu M Kurosawa H Yoshida S Comparative study of regulatory T cell function of human CD25CD4 T cells from thymocytes, cord blood, and adult peripheral blood. Clin Dev Immunol (2008) 2008:305859. 10.1155/2008/305859 18815628 PMC2547481

[13] Wing K Ekmark A Karlsson H Rudin A Suri-Payer E. Characterization of human CD25+ CD4+ T cells in thymus, cord and adult blood. Immunology (2002) 106(2):190–9. 10.1046/j.1365-2567.2002.01412.x 12047748 PMC1782718

[14] Wing K Larsson P Sandstrom K Lundin SB Suri-Payer E Rudin A. CD4+ CD25+ FOXP3+ regulatory T cells from human thymus and cord blood suppress antigen-specific T cell responses. Immunology (2005) 115(4):516–25. 10.1111/j.1365-2567.2005.02186.x 16011520 PMC1782183

[15] Belkaid Y Piccirillo CA Mendez S Shevach EM Sacks DL. CD4+CD25+ regulatory T cells control *Leishmania major* persistence and immunity. Nature (2002) 420(6915):502–7. 10.1038/nature01152 12466842

[16] Ndure J Flanagan KL. Targeting regulatory T cells to improve vaccine immunogenicity in early life. Front Microbiol (2014) 5:477. 10.3389/fmicb.2014.00477 25309517 PMC4161046

[17] Noho-Konteh F Adetifa JU Cox M Hossin S Reynolds J Le MT Sex-differential non-vaccine specific immunological effects of diphtheria-tetanus-pertussis and measles vaccination. Clin Infect Dis (2016) 63(9):1213–26. 10.1093/cid/ciw492 27436422

[18] Whittle HC Campbell H Rahman S Armstrong JR. Antibody persistence in Gambian children after high-dose Edmonston-Zagreb measles vaccine. Lancet (1990) 336(8722):1046–8. 10.1016/0140-6736(90)92501-8 1977029

[19] van Gageldonk PG van Schaijk FG van der Klis FR Berbers GA. Development and validation of a multiplex immunoassay for the simultaneous determination of serum antibodies to *Bordetella pertussis*, diphtheria and tetanus. J Immunol Methods (2008) 335(1–2):79–89. 10.1016/j.jim.2008.02.018 18407287

[20] Lahl K Sparwasser T. In vivo depletion of FoxP3+ Tregs using the DEREG mouse model. Methods Mol Biol (2011) 707:157–72. 10.1007/978-1-61737-979-6_10 21287334

[21] Klages K Mayer CT Lahl K Loddenkemper C Teng MW Ngiow SF Selective depletion of Foxp3+ regulatory T cells improves effective therapeutic vaccination against established melanoma. Cancer Res (2010) 70(20):7788–99. 10.1158/0008-5472.CAN-10-1736 20924102

[22] Mattarollo SR Steegh K Li M Duret H Foong Ngiow S Smyth MJ. Transient Foxp3(+) regulatory T-cell depletion enhances therapeutic anticancer vaccination targeting the immune-stimulatory properties of NKT cells. Immunol Cell Biol (2013) 91(1):105–14. 10.1038/icb.2012.58 23090488

[23] Moore AC Gallimore A Draper SJ Watkins KR Gilbert SC Hill AV. Anti-CD25 antibody enhancement of vaccine-induced immunogenicity: increased durable cellular immunity with reduced immunodominance. J Immunol (2005) 175(11):7264–73. 10.4049/jimmunol.175.11.7264 16301631

[24] Ho P Wei X Seah GT. Regulatory T cells induced by *Mycobacterium chelonae* sensitization influence murine responses to bacille Calmette-Guerin. J Leukoc Biol (2010) 88(6):1073–80. 10.1189/jlb.0809582 20651297

[25] Macatangay BJ Szajnik ME Whiteside TL Riddler SA Rinaldo CR. Regulatory T cell suppression of Gag-specific CD8 T cell polyfunctional response after therapeutic vaccination of HIV-1-infected patients on ART. PLoS One (2010) 5(3):e9852. 10.1371/journal.pone.0009852 20352042 PMC2844424

[26] Burl S Adetifa UJ Cox M Touray E Ota MO Marchant A Delaying bacillus Calmette-Guerin vaccination from birth to 4 1/2 months of age reduces postvaccination Th1 and IL-17 responses but leads to comparable mycobacterial responses at 9 months of age. J Immunol (2010) 185(4):2620–8. 10.4049/jimmunol.1000552 20644160

[27] de Cassan SC Pathan AA Sander CR Minassian A Rowland R Hill AV Investigating the induction of vaccine-induced Th17 and regulatory T cells in healthy, *Mycobacterium bovis* BCG-immunized adults vaccinated with a new tuberculosis vaccine, MVA85A. Clin Vaccine Immunol (2010) 17(7):1066–73. 10.1128/CVI.00047-10 20484570 PMC2897259

[28] Fletcher HA Pathan AA Berthoud TK Dunachie SJ Whelan KT Alder NC Boosting BCG vaccination with MVA85A down-regulates the immunoregulatory cytokine TGF-beta1. Vaccine (2008) 26(41):5269–75. 10.1016/j.vaccine.2008.07.040 18682270 PMC2631167

[29] Cook IF. Sexual dimorphism of humoral immunity with human vaccines. Vaccine (2008) 26(29–30):3551–5. 10.1016/j.vaccine.2008.04.054 18524433

[30] Klein SL Poland GA Personalized vaccinology: one size and dose might not fit both sexes. Vaccine (2013) 31(23):2599–600. 10.1016/j.vaccine.2013.02.070 23579257

[31] Furman D Hejblum BP Simon N Jojic V Dekker CL Thiebaut R Systems analysis of sex differences reveals an immunosuppressive role for testosterone in the response to influenza vaccination. Proc Natl Acad Sci U S A (2014) 111(2):869–74. 10.1073/pnas.1321060111 24367114 PMC3896147

[32] Klein SL Flanagan KL Sex differences in the immune response. Nat Rev Immunol (2016) 16(10):626–38. 10.1038/nri.2016.90 27546235

[33] Flanagan KL van Crevel R Curtis N Shann F Levy O Heterologous (“nonspecific”) and sex-differential effects of vaccines: epidemiology, clinical trials, and emerging immunologic mechanisms. Clin Infect Dis (2013) 57(2):283–9. 10.1093/cid/cit209 23572484 PMC3689344

[34] Flanagan KL Plebanski M Sex-differential heterologous (non-specific) effects of vaccines: an emerging public health issue that needs to be understood and exploited. Expert Rev Vaccines (2016) 16(1):5–13. 10.1080/14760584.2016.1203260 27362915

[35] Dunachie SJ Berthoud T Keating SM Hill AV Fletcher HA. MIG and the regulatory cytokines IL-10 and TGF-β1 correlate with malaria vaccine immunogenicity and efficacy. PLoS One (2010) 5(9):e12557. 10.1371/journal.pone.0012557 20838432 PMC2933226

[36] Lim HW Hillsamer P Banham AH Kim CH Direct suppression of B cells by CD4^+^CD25^+^ regulatory T cells. J Immunol (2005) 175(7):4180–3. 10.4049/jimmunol.175.7.4180 16177055

[37] Lim HW Hillsamer P Kim CH. Regulatory T cells can migrate to follicles upon T cell activation and suppress GC-Th cells and GC-Th cell-driven B cell responses. J Clin Invest (2004) 114(11):1640–9. 10.1172/JCI122325 15578096 PMC529283

[38] Perret R Sierro SR Botelho NK Corgnac S Donda A Romero P. Adjuvants that improve the ratio of antigen-specific effector to regulatory T cells enhance tumor immunity. Cancer Res (2013) 73(22):6597–608. 10.1158/0008-5472.CAN-13-0875 24048821

[39] Bayry J. Regulatory T cells as adjuvant target for enhancing the viral disease vaccine efficacy. Virusdisease (2014) 25(1):18–25. 10.1007/s13337-013-0187-3 24426307 PMC3889236

[40] Voo KS Bover L Harline ML Vien LT Facchinetti V Arima K Antibodies targeting human OX40 expand effector T cells and block inducible and natural regulatory T cell function. J Immunol (2013) 191(7):3641–50. 10.4049/jimmunol.1202752 24014877 PMC3812678

[41] Barbon CM Yang M Wands GD Ramesh R Slusher BS Hedley ML Consecutive low doses of cyclophosphamide preferentially target Tregs and potentiate T cell responses induced by DNA PLG microparticle immunization. Cell Immunol (2010) 262(2):150–61. 10.1016/j.cellimm.2010.02.007 20206921

[42] Le DT Jaffee EM. Regulatory T-cell modulation using cyclophosphamide in vaccine approaches: a current perspective. Cancer Res (2012) 72(14):3439–44. 10.1158/0008-5472.CAN-11-3912 22761338 PMC3399042

[43] Tan C Reddy V Dannull J Ding E Nair SK Tyler DS Impact of anti-CD25 monoclonal antibody on dendritic cell-tumor fusion vaccine efficacy in a murine melanoma model. J Transl Med (2013) 11:148. 10.1186/1479-5876-11-148 23768240 PMC3691646

[44] Keenan BP Saenger Y Kafrouni MI Leubner A Lauer P Maitra A A *Listeria* vaccine and depletion of T-regulatory cells activate immunity against early stage pancreatic intraepithelial neoplasms and prolong survival of mice. Gastroenterology (2014) 146(7):1784–94.e6. 10.1053/j.gastro.2014.02.055 24607504 PMC4035450

[45] Goebel J Stevens E Forrest K Roszman TL. Daclizumab (Zenapax) inhibits early interleukin-2 receptor signal transduction events. Transpl Immunol (2000) 8(3):153–9. 10.1016/S0966-3274(00)00021-6 11147695

[46] Kohm AP McMahon JS Podojil JR Begolka WS DeGutes M Kasprowicz DJ Cutting edge: anti-CD25 monoclonal antibody injection results in the functional inactivation, not depletion, of CD4+CD25+ T regulatory cells. J Immunol (2006) 176(6):3301–5. 10.4049/jimmunol.176.6.3301 16517695

[47] Rech AJ Mick R Martin S Recio A Aqui NA Powell DJ Jr CD25 blockade depletes and selectively reprograms regulatory T cells in concert with immunotherapy in cancer patients. Sci Transl Med (2012) 4(134):134ra62. 10.1126/scitranslmed.3003330 22593175 PMC4425934

[48] Rech AJ Vonderheide RH. Clinical use of anti-CD25 antibody daclizumab to enhance immune responses to tumor antigen vaccination by targeting regulatory T cells. Ann N Y Acad Sci (2009) 1174:99–106. 10.1111/j.1749-6632.2009.04939 19769742

[49] Peggs KS Quezada SA Chambers CA Korman AJ Allison JP. Blockade of CTLA-4 on both effector and regulatory T cell compartments contributes to the antitumor activity of anti-CTLA-4 antibodies. J Exp Med (2009) 206(8):1717–25. 10.1084/jem.20082492 19581407 PMC2722174

